# Ecdysone-dependent feedback regulation of prothoracicotropic hormone controls the timing of developmental maturation

**DOI:** 10.1242/dev.188110

**Published:** 2020-07-24

**Authors:** Christian F. Christensen, Takashi Koyama, Stanislav Nagy, E. Thomas Danielsen, Michael J. Texada, Kenneth A. Halberg, Kim Rewitz

**Affiliations:** Department of Biology, University of Copenhagen, 2100 Copenhagen O, Denmark; Department of Biology, University of Copenhagen, 2100 Copenhagen O, Denmark; Department of Biology, University of Copenhagen, 2100 Copenhagen O, Denmark; Department of Biology, University of Copenhagen, 2100 Copenhagen O, Denmark; Department of Biology, University of Copenhagen, 2100 Copenhagen O, Denmark; Department of Biology, University of Copenhagen, 2100 Copenhagen O, Denmark; Department of Biology, University of Copenhagen, 2100 Copenhagen O, Denmark

**Keywords:** *Drosophila*, Ecdysone, Maturation, Prothoracicotropic, Ptth, Steroid

## Abstract

The activation of a neuroendocrine system that induces a surge in steroid production is a conserved initiator of the juvenile-to-adult transition in many animals. The trigger for maturation is the secretion of brain-derived neuropeptides, yet the mechanisms controlling the timely onset of this event remain ill-defined. Here, we show that a regulatory feedback circuit controlling the *Drosophila* neuropeptide Prothoracicotropic hormone (PTTH) triggers maturation onset. We identify the Ecdysone Receptor (EcR) in the PTTH-expressing neurons (PTTHn) as a regulator of developmental maturation onset. Loss of *EcR* in these PTTHn impairs PTTH signaling, which delays maturation. We find that the steroid ecdysone dose-dependently affects *Ptth* transcription, promoting its expression at lower concentrations and inhibiting it at higher concentrations. Our findings indicate the existence of a feedback circuit in which rising ecdysone levels trigger, via EcR activity in the PTTHn, the PTTH surge that generates the maturation-inducing ecdysone peak toward the end of larval development. Because steroid feedback is also known to control the vertebrate maturation-inducing hypothalamic-pituitary-gonadal axis, our findings suggest an overall conservation of the feedback-regulatory neuroendocrine circuitry that controls the timing of maturation initiation.

## Teaser

**Summary:** Feedback activation of the neuropeptide PTTH induces a maturation-inducing surge in steroid production that regulates the timing of the juvenile-to-adult transition in *Drosophila*.

## INTRODUCTION

The activation of a neuroendocrine axis leading to the production of steroid hormones is a conserved trigger of maturation onset in animals (Rewitz et al., 2013; Sisk and Foster, 2004; Tennessen and Thummel, 2011). In vertebrates, neuronal gonadotropin-releasing hormone (GnRH) secretion awakens the hypothalamic-pituitary-gonadal (HPG) axis leading to the production of sex steroids at puberty. Similarly, the insect neuropeptide Prothoracicotropic hormone (PTTH) induces steroidogenesis that drives the juvenile-to-adult transition. Multiple signals converge on these neuroendocrine axes to couple the timing of maturation with external and internal stimuli including nutritional and metabolic states (Mirth and Riddiford, 2007; Navarro et al., 2007). In humans, the prevalence of childhood obesity is believed to be a key factor in the growing incidence of early puberty, which affects growth and final body size (Ahmed et al., 2009; Kaplowitz, 2008). In *Drosophila* and other insects, the onset of maturation is governed by a nutritional checkpoint associated with the attainment of critical body size or weight, after which maturation will occur irrespective of nutritional intake (Edgar, 2006; Mirth and Riddiford, 2007). Thus, maturation is not initiated until the juvenile has accumulated sufficient energy and material to ensure successful maturation and adult fitness.

PTTH is produced by a pair of neurosecretory cells (PTTHn) in each brain hemisphere (McBrayer et al., 2007). The axons of these neurons terminate on the steroid-producing cells of the prothoracic gland (PG) (McBrayer et al., 2007), forming a neuroendocrine circuit functionally similar to the mammalian HPG axis. PTTH released here binds to Torso, its cognate receptor tyrosine kinase, initiating a mitogen-activated protein kinase (MAPK) cascade that leads to the production of the steroid ecdysone (Rewitz et al., 2009). Ecdysone is then released from the PG cells through a regulated vesicle-mediated process (Yamanaka et al., 2015) and, in peripheral tissues, is converted into its more potent form, 20-hydroxyecdysone (20E) (Rewitz et al., 2013). Actions of 20E are mediated by its binding to the nuclear Ecdysone Receptor (EcR), which, in a heterodimeric complex with Ultraspiracle (Usp), induces time- and tissue-specific transcriptional responses that initiate metamorphosis, a transition from the juvenile larval stage to the adult similar to mammalian puberty (King-Jones and Thummel, 2005).

Uncovering the signals that affect the production and release of PTTH is key to understanding how maturation timing is controlled. *Drosophila* Insulin-like Peptide 8 (DILP8; Ilp8 – FlyBase) secreted by adult precursor tissues developing within the larva prevents maturation onset until these tissues are sufficiently developed, through regulation of neurons that inhibit the PTTHn (Colombani et al., 2015, 2012; Garelli et al., 2012, 2015; Vallejo et al., 2015). The neuropeptide Pigment-Dispersing Factor (PDF) (McBrayer et al., 2007), a signal associated with the circadian clock, also regulates PTTH, suggesting that the fly maturation pathway, like the mammalian HPG axis, is under photoperiodic control (Walton et al., 2011). In mammals, the peptide kisspeptin (KISS1) and its receptor GPR54 (KISS1R) are key triggers of puberty onset that regulate GnRH secretion. Similarly, the *Drosophila* KISS1 ortholog, Allatostatin A (AstA), and its receptor regulate maturation onset via control of PTTH signaling (Deveci et al., 2019; Pan and O'Connor, 2019). Furthermore, the HPG axis is under feedback control, whereby gonadal steroids act on the upstream neuroendocrine components to regulate signaling through this axis (Acevedo-Rodriguez et al., 2018). In *Drosophila*, ecdysone provides positive and negative feedback on the PG in a classical feedback loop to shape the pulses of ecdysone necessary to drive the genetic programs underlying the juvenile-to-adult transition (Moeller et al., 2013). However, whether feedback also regulates the neurocircuitry that triggers these steroid pulses in animals, and thus the overall timing of the juvenile-to-adult transition, is presently unknown.

We report here the identification of *EcR* and *usp* in an RNAi-based screen for regulators of PTTH production or release. We find that ecdysone-mediated feedback via EcR in the PTTHn drives the pupariation-triggering PTTH surge, thereby determining the timing of maturation. Under rising ecdysone levels, EcR mediates *Ptth* transcriptional upregulation, leading to the steep rise in PTTH prior to metamorphosis. This generates a PTTH surge that induces a high-level ecdysone peak that initiates maturation; high ecdysone levels subsequently feed back negatively to suppress PTTH production. Mammalian gonadal steroids act in positive and negative feedback loops to modulate the HPG axis (Acevedo-Rodriguez et al., 2018). Our results show that the developmental transition to adulthood in *Drosophila* is similarly controlled by positive and negative feedback mechanisms that modulate the PTTHn, suggesting that neuroendocrine feedback control of developmental maturation is evolutionarily ancient.

## RESULTS

### EcR induces maturation onset through positive regulation of the PTTHn

**Fig. 1.**
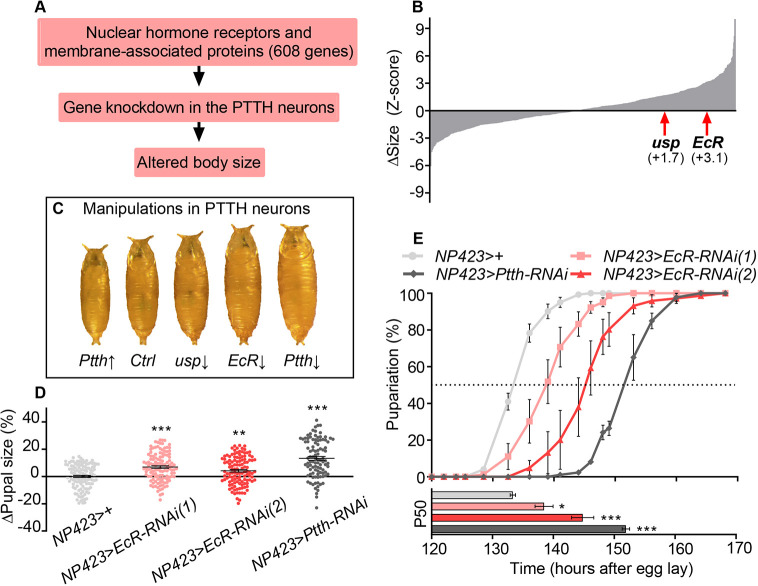
**Screening for regulators of PTTH identifies the Ecdysone Receptor (EcR) complex.** (A) Outline of the PTTHn screen for factors regulating growth. Gene expression was knocked down in the PTTHn using the strong *NP423-GAL4* (*NP432>*) driver, and pupal size was measured. In *Drosophila*, growth is restricted to the larval stage, and pupal size thus determines final adult size. (B) Pupal-size distribution from the screen, presented as a Z score (standard deviations from the mean of all RNAi lines). RNAi against *EcR* or its partner *ultraspiracle* (*usp*) led to increased pupal size (+3.1 and +1.7 s.d.). (C) Images of representative pupae of animals with NP423-driven overexpression of *UAS-Ptth* (*Ptth* ↑), *UAS-usp-RNAi* (*usp* ↓), *UAS-EcR-RNAi* (*EcR* ↓) or *UAS-Ptth-RNAi* (*Ptth* ↓). (D) *EcR* knockdown using *NP423>* with two independent RNAi lines led to increased pupal size, similar to RNAi against *Ptth*. (E) RNAi knockdown of *EcR* or *Ptth* delays pupariation, prolonging the feeding stage of development. Top: curve showing the fraction of pupated animals over time; bottom: the corresponding 50%-pupariated ‘P50’ times. Statistics: one-way ANOVA with Dunnett's multiple comparison test; *P<0.05; **P<0.01; ***P<0.001.

PTTH release is thought to be the main trigger of the juvenile-adult transition in insects (Rewitz et al., 2013). Loss of PTTH extends the larval period of feeding and growth, leading to increased pupal and adult size. To identify signals controlling maturation onset, upstream of PTTH, we performed an RNAi-based pupal-size screen targeting 608 membrane-associated proteins (known or potential receptors) and transcription factors in the PTTHn (Fig. 1A, Table S1). In this screen, expression of RNAi constructs (along with the RNA-processing enzyme Dicer-2) was driven in the PTTHn by *NP423-GAL4* (*NP423>*) (Deveci et al., 2019; Gong et al., 2010; Yamanaka et al., 2013). Among the strongest hits was *EcR*; knockdown of this gene, or of *usp*, which encodes a transcriptional co-factor of EcR, in the PTTHn led to larval overgrowth and thus to increased pupal size (Fig. 1B). To verify these findings, we reduced *EcR* expression in the larval PTTHn and measured developmental timing and final body size. Knockdown using two independent RNAi lines (#1 and #2) confirmed that loss of *EcR* in the PTTHn causes developmental delay and overgrowth, like reduced expression of *Ptth* itself (Fig. 1C-E). This suggested that EcR might act in the PTTHn as a positive regulator of PTTH production and release. 

**Fig. 2.**
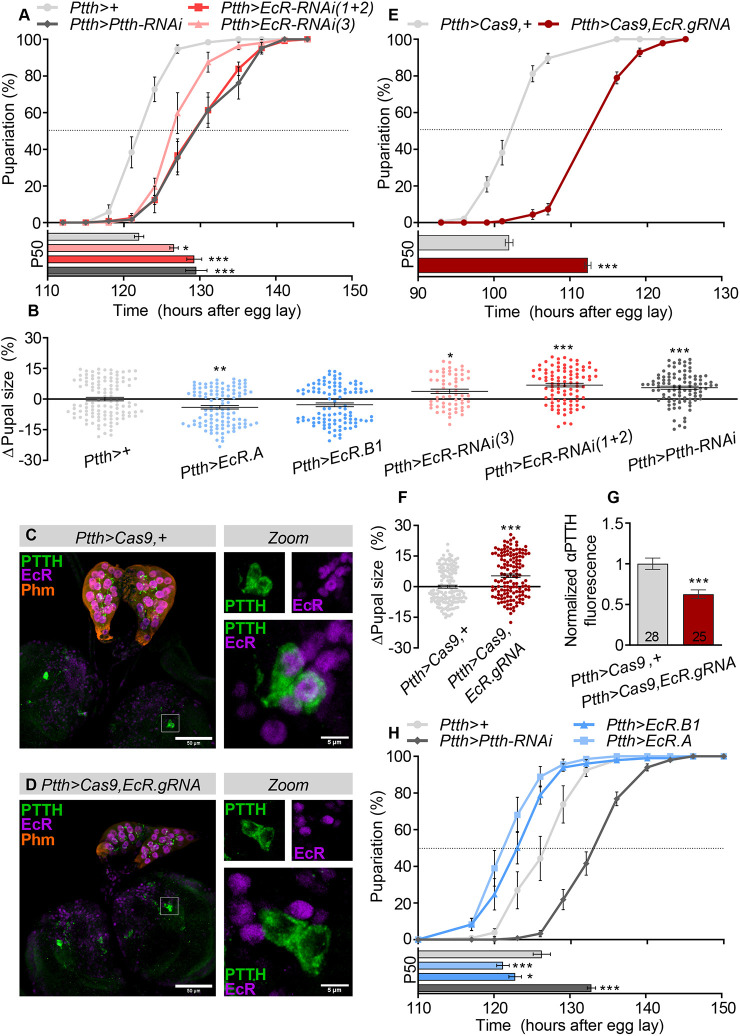
**EcR activity in the PTTHn controls the onset of maturation.** (A) RNAi-induced knockdown of *EcR* with RNAi lines #1 and #2 combined or a third (single) RNAi line (#3) against *EcR* using the specific *Ptth-GAL4* (*Ptth*>) driver delayed pupariation to a similar extent as knockdown of *Ptth*, recapitulating the results seen with the strong *NP423>* driver. Top: pupariation over time; bottom: P50 values. (B) PTTHn-specific manipulations of *EcR* expression alter pupal size. Overexpression of the A isoform of EcR led to reduced pupal size, whereas RNAi against *EcR* or *Ptth* increased pupal size. Bimodal distributions reflect sexual size dimorphism. (C,D) At 96 h AEL at 29°C, EcR immunostaining is present in the nuclei of PTTHn and of neighboring neurons in the control genotype (*Ptth-GAL4, UAS-Cas9*,+; *Ptth>Cas9*,+) for PTTHn-specific CRISPR/Cas9-induced *EcR* deletion (C). EcR immunostaining is eliminated specifically in the PTTHn in animals expressing both Cas9 and the *EcR* guide-RNA construct in these cells, illustrating the cell-specificity of the deletion (D). Scale bars: 50 μm (main panels); 5 μm (insets). (E,F) CRISPR/Cas9-mediated disruption of the *EcR* locus in the PTTHn leads to (E) developmental delay and (F) a corresponding pupal size increase (at 30°C for stronger GAL4 and Cas9 activity; note the temperature-induced growth acceleration of the control animals, compared with the data shown in A). (G) *EcR* disruption in the PTTHn leads to reduced PTTH immunostaining in these cells. The number (*n*) of individual neurons measured is indicated on the bars. Intensity values are normalized against the average value of controls. (H) Overexpression of EcR A or B1 isoforms in the PTTHn leads to accelerated development and premature metamorphosis. Statistics: one-way ANOVA with Dunnett's multiple comparisons or an unpaired two-tailed *t*-test for pairwise comparison; *P<0.05; **P<0.01; ***P<0.001.

Next, we used the *Ptth-GAL4* (*Ptth>*) driver, a weaker but PTTHn-specific line, to confirm that these effects were caused by knockdown of *EcR* in the PTTHn. Because of *Ptth-GAL4*’s relative weakness, we recombined *EcR-RNAi* constructs #1 and #2 to increase the strength of RNAi-mediated knockdown. Animals expressing *Ptth>EcR-RNAi-1+2* exhibited delayed pupariation and increased pupal size comparable to knockdown of *Ptth* itself, and we re-confirmed these effects using a third independent *EcR-RNAi* line, #3 (Fig. 2A,B). All further *EcR-RNAi* experiments used RNAi lines #1 and #2 combined. To further attribute these defects to PTTHn-specific EcR deficiency, we disrupted the *EcR* gene in only these cells using tissue-specific somatic CRISPR/Cas9 gene editing. We generated a transgenic UAS-regulated construct that expresses a pair of guide RNAs (gRNAs) targeting exon 3 of the *EcR* gene, which encodes the DNA-binding domain of EcR and is shared between all its isoforms. By expressing this construct along with *UAS-Cas9* under *Ptth>* control (at 29°C to boost the activity of the GAL4 transcription factor and the Cas9 enzyme), we disrupted the *EcR* locus specifically in the PTTHn. To assess the efficacy of this setup, we immunostained brains from larvae at 96 h AEL (after egg laying) using antibodies against the PTTH neuropeptide, EcR, and the ecdysone-biosynthetic enzyme Phantom (Phm) to label the PG (Rewitz et al., 2006; Warren et al., 2004). No anti-EcR signal was visible in the PTTHn of knockout animals, whereas clear anti-EcR signal was present in the PTTHn nuclei of controls, and in neighboring cells in both controls and *EcR* knockouts (Fig. 2C,D). Although the CRISPR/Cas9 system has induced cytotoxicity in some reports, we did not observe any morphological abnormalities in the PTTHn or their projections to the PG. Next, to investigate how this manipulation affected PTTHn activity, we measured developmental timing and final body size. PTTHn-specific *EcR* disruption significantly delayed pupariation and resulted in larger pupae, compared with controls (Fig. 2E,F). Furthermore, PTTH levels (anti-PTTH staining intensity in the PTTHn cell body) were significantly reduced in *EcR* knockouts (Fig. 2G). Together, these data support a role for EcR as an inducer of PTTH expression. We therefore next overexpressed *EcR* in the PTTHn to analyze whether this might lead to a precocious juvenile-adult transition. Indeed, overexpressing *EcR.A* or *EcR.B1* variants resulted in advanced pupariation onset, thereby shortening the larval growth period and reducing pupal size (Fig. 2B,H), consistent with a positive effect of EcR on PTTHn activity. 

### EcR induces the PTTH surge prior to the juvenile-adult transition

**Fig. 3.**
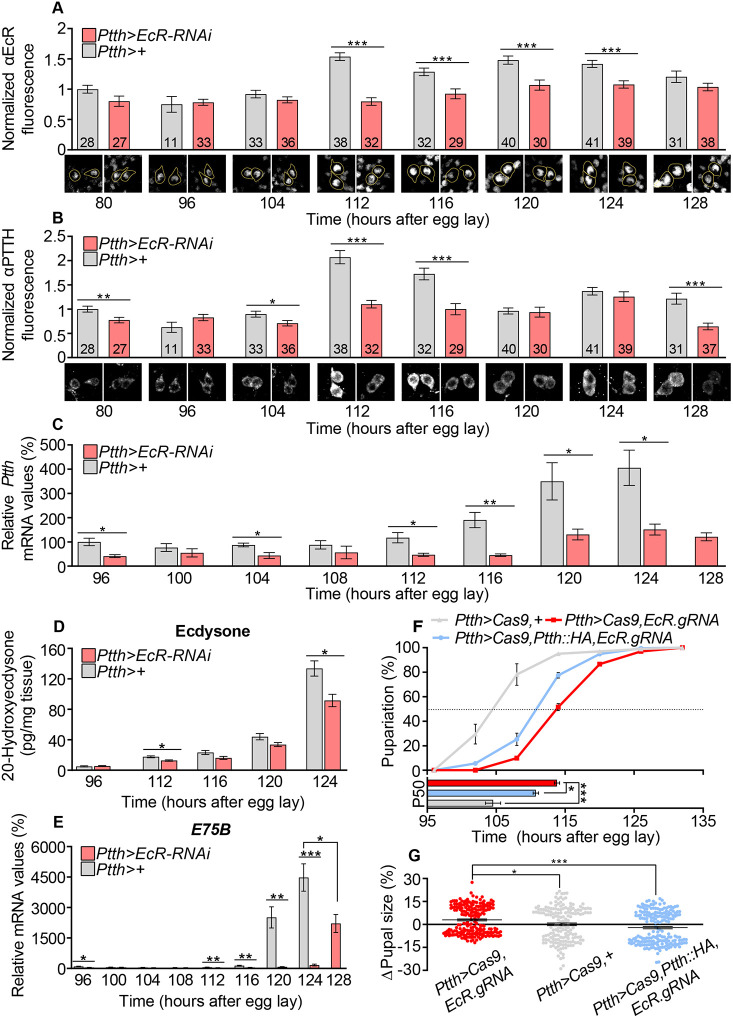
**EcR is required in the PTTHn to induce PTTH toward the end of larval development.** (A) Late-third-instar upregulation of EcR in nuclei of PTTHn is attenuated by *EcR* knockdown. PTTHn were identified by PTTH immunostaining (the same cells are shown in B) and are circled in the representative image pairs below. (B) *EcR* knockdown in the PTTHn prevents the late-third-instar increase in PTTH expression. PTTH immunostaining intensity increases in control animals at 112 h AEL, whereas this increase does not take place in *EcR*-knockdown animals. Top: quantification of PTTH immunostaining intensity; bottom: representative images. In A and B, the number (*n*) of individual neurons measured at each time point is indicated in bars. All intensity values are normalized against the average value of controls at 80 h for each channel. (C) *EcR* knockdown in the PTTHn reduces *Ptth* expression at all time points and prevents or diminishes the upregulation of its expression toward the end of larval development (a 128-h time point is included for knockdown animals to illustrate the lack of further increase). (D) The late-third-instar peak in 20-hydroxyecdysone levels is reduced or delayed by *EcR* knockdown in the PTTHn. (E) The corresponding expression increase in the ecdysone-responsive proxy gene *E75B* is delayed in *EcR*-knockdown animals; again, a 128-h measurement is included for the developmentally delayed knockdown animals, illustrating that the increase in *E75B* expression eventually appears, although it is delayed by 8-12 h. (F,G) Rescue of developmental timing and growth phenotypes induced by CRISPR/Cas9-mediated *EcR* knockout in the PTTHn by *Ptth* overexpression. Statistics: unpaired two-tailed *t*-test for pairwise comparison; *P<0.05; **P<0.01; ***P<0.001.

Because EcR induces pupariation through its actions in the PTTHn, we investigated EcR levels in these cells during the juvenile-adult transition. We found that EcR is present in PTTHn nuclei from early L3 (80 h AEL) throughout the L3 stage, increasing in abundance at the onset of the wandering stage (112 h AEL), when ecdysone levels rise prior to pupariation (Fig. 3A). Knockdown of *EcR* in the PTTHn attenuated this EcR rise, confirming that *EcR* expression is reduced by the RNAi, albeit not eliminated as with CRISPR-induced deletion (Fig. 2C,D). 

As *Ptth* is transcriptionally upregulated to induce the ecdysone pulse that triggers maturation (McBrayer et al., 2007), we analyzed whether EcR is required in the PTTHn for this upregulation. Anti-PTTH immunostaining intensity in cell bodies of the PTTHn displayed a dynamic profile in control animals, with a strong increase at 112 h AEL that coincided with the increase in EcR abundance (Fig. 3B), consistent with EcR being responsible for PTTH upregulation at this time. PTTHn-specific *EcR* knockdown markedly changed this pattern, eliminating the increase in PTTH intensity at 112 and 116 h AEL observed in control animals. Although a reduction in PTTH staining could potentially be explained by increased secretion, this would be inconsistent with these animals' delayed development. Thus, this altered profile suggests that EcR positively regulates PTTH production, consistent with the results showing that EcR expression in the PTTHn correlates with pupariation advance. Because EcR is a transcriptional regulator, we asked whether EcR is required for transcriptional upregulation of *Ptth* during the late L3 stage by performing a temporal gene-expression analysis of larvae expressing *EcR-RNAi* in the PTTHn throughout the second half of L3. Consistent with previous findings (McBrayer et al., 2007), in control animals, *Ptth* expression underwent a dramatic upregulation from about 12 h prior to pupariation (112-124 h AEL) until the onset of metamorphosis (Fig. 3C). *EcR* knockdown significantly reduced *Ptth* expression across the time series and prevented its pre-wandering enhancement, indicating that EcR is required for the late-larval transcriptional upregulation of *Ptth*.

### Loss of *EcR* in the PTTHn impairs the steroid increase that triggers maturation

PTTH signaling stimulates the production of ecdysone in the PG by inducing transcriptional upregulation of the ecdysone-biosynthetic Halloween genes (McBrayer et al., 2007; Shimell et al., 2018). To examine the effects of EcR in the PTTHn on ecdysone production, we first analyzed the expression of these genes in the PG. Expression of *spookier*, *phantom*, *disembodied* and *shadow* are all upregulated in the PG in response to PTTH/Torso signaling. The enzyme Shade mediates the conversion of ecdysone to 20E in peripheral tissues, and its expression is therefore not regulated by PTTH signaling (McBrayer et al., 2007; Ou et al., 2016). Consistent with the requirement of EcR for inducing PTTH production, we found that the ecdysone-synthesis genes were not properly upregulated in *Ptth>EcR-RNAi* animals, whereas levels of *shade* were unaffected (Fig. S1A-E). This is consistent with a model in which the delay observed in *EcR*-knockdown animals is caused by a failure to upregulate PTTH towards the end of larval development, thus impairing the ecdysone biosynthetic pathway. To determine whether this is the case, we analyzed ecdysone levels directly by enzyme-linked immunosorbent assay (ELISA) and found reduced levels of ecdysone in animals with *EcR* knockdown in the PTTHn towards the end of larval development (Fig. 3D), indicating that they do not produce a proper maturation-promoting ecdysone pulse. As a result, expression of the ecdysone-inducible genes *E75A* and *E75B*, which serve as proxies for the ecdysone titer, show a significantly reduced and delayed rise in *Ptth>EcR-RNAi* animals (Fig. 3E, Fig. S2). These data suggest that EcR stimulates *Ptth* transcription to generate the PTTH surge that initiates metamorphosis. We therefore examined whether the developmental-delay phenotype caused by knockout of *EcR* in the PTTHn could be rescued by simultaneous overexpression of *Ptth*. Indeed, *Ptth* overexpression in the PTTHn of animals with CRISPR/Cas9-mediated knockout of *EcR* in these same neurons partially rescued the delayed pupariation and completely rescued their overgrowth, showing that the phenotype is caused by lack of PTTH (Fig. 3F,G). Together, our results suggest that EcR acts as a positive regulator of *Ptth* expression, just as steroids modulate the mammalian HPG axis, and that feedback through the steroid ecdysone is a key trigger of the neuroendocrine cascade that drives maturation onset in *Drosophila*.

### Initiation of maturation is triggered by feedback that activates PTTH

**Fig. 4.**
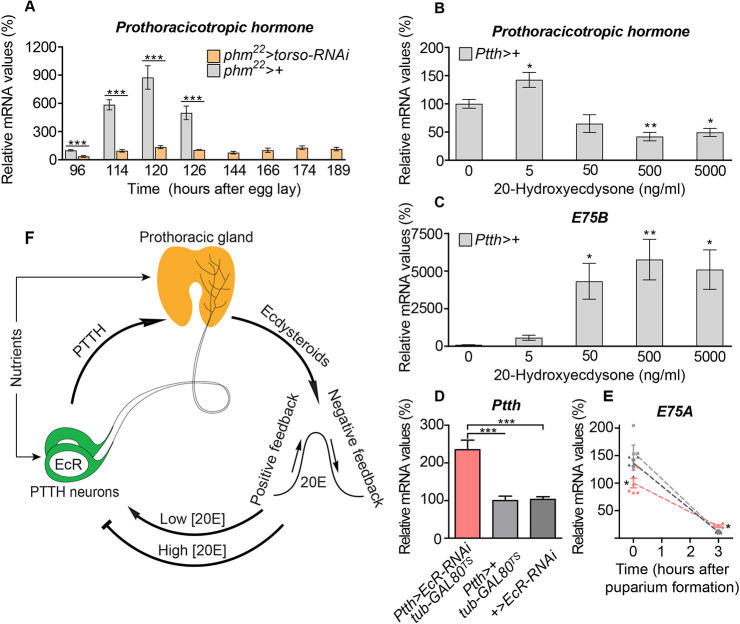
***Ptth* is upregulated by ecdysone-mediated feedback at the onset of maturation.** (A) The larval peak of *Ptth* expression observed in the control genotype does not appear in *phm*>*torso-RNAi* animals, indicating feedback from ecdysone produced by the PG to the PTTHn. (B,C) Expression of (B) *Ptth* in *ex-vivo* brains is increased by low levels of 20-hydroxyecdysone (20E, the more active form of ecdysone), but inhibited by larger concentrations, whereas expression of (C) the EcR-regulated proxy gene *E75B* increases in *ex vivo* cultured brains with increasing concentration of 20E in the medium, indicating biphasicity of ecdysone response at the *Ptth* locus. (D) RNAi-induced knockdown of *EcR* beginning 10 h before pupariation leads to increased *Ptth* transcription 3 h post-pupariation, consistent with a lack of EcR-mediated inhibition. (E) Levels of the ecdysone-induced transcript *E75A* are higher at the time of pupariation in temperature-induced *EcR*-knockdown animals than in controls, but after 3 h, *E75A* levels have fallen to a lower level in these animals than in controls, suggesting increased or prolonged ecdysone levels, consistent with loss of EcR-mediated *Ptth* inhibition. Colors in E are the same as in D. Statistics: one-way ANOVA with Dunnett's multiple comparisons or an unpaired two-tailed *t*-test for pairwise comparison; *P<0.05; **P<0.01; ***P<0.001. (F) Graphical summary of the model presented here. A small rise in ecdysone production by the PG feeds back in an EcR-dependent manner in the PTTHn to drive the metamorphosis-inducing surge of PTTH release and ecdysone production; high ecdysone levels at the peak of the surge in turn inhibit further PTTH expression.

EcR and 20E sit atop a large network of transcriptional regulators (King-Jones and Thummel, 2005). Our results show that EcR is required in the PTTHn for *Ptth* upregulation, suggesting a model in which PTTH or other factors induce a small ecdysone rise that acts via EcR in the PTTHn to increase PTTH production, leading to the activation of the metamorphosis-initiating neuroendocrine cascade. In this model, *in vivo* ecdysone manipulations should feed back to affect PTTH levels. Thus, we reduced ecdysone synthesis by silencing *torso*, which encodes the PTTH receptor, in the PG [PG-specific *phm-GAL4* (*phm*>) driving *UAS-torso-RNAi* (*torso-RNAi*): *phm>torso-RNAi*]. As ecdysone synthesis is downregulated in these animals, their larval feeding stage was prolonged, resulting in overgrowth and increased pupal size (Fig. S3A). Next, we measured *Ptth* levels during L3 prior to the onset of pupariation. In controls, *Ptth* expression increased as expected from 96 h AEL to 120 h AEL (Fig. 4A). By contrast, when ecdysone synthesis was reduced via *phm>torso-RNAi*, *Ptth* expression remained uninduced, even when these animals eventually pupariated (at 189 h AEL). Expression of the PG-specific ecdysone-biosynthetic gene *disembodied* and the ecdysone-induced gene *E75B* remained low in *phm*>*torso-RNAi* animals, confirming reduced ecdysone synthesis (Fig. S3B,C). Thus, blocking ecdysone synthesis in the PG prevents the pre-metamorphic upregulation of *Ptth*. 

We next dissected larval brains, preserving PTTHn projections to the ring gland, which contains the PG, and cultured them *ex vivo* for 6 h in media containing 20E. Consistent with our model, 20E at 5 ng/ml (in the low physiological concentration range occurring during L3, prior to the large pre-metamorphosis pulse) increased *Ptth* expression (Fig. 4B). This suggests that pre-pulse ecdysone levels suffice to awaken the neuroendocrine system through *Ptth* upregulation, which then induces the steroid pulse initiating maturation. Interestingly, higher concentrations of 20E inhibited *Ptth* transcription, inconsistent with a solely positive role for 20E/EcR. *Ptth-*inhibitory 20E concentrations (500 ng/ml) correspond to high physiological levels that occur during the large pupariation-associated ecdysone pulse. Consistent with this, these concentrations induced *E75B* upregulation (Fig. 4C), which occurs during this maturation-inducing pulse. We further tested the potential of negative feedback downregulation of PTTH following pupariation when the ecdysone titer falls rapidly. To do this, we induced RNAi-mediated knockdown of *EcR* 10 h before pupariation, at a time when *Ptth* expression has already been upregulated, to prevent interference with the positive effect of EcR on the pupariation-triggering rise in *Ptth* expression. This manipulation led to increased *Ptth* expression 3 h following pupariation (Fig. 4D), suggesting that EcR is required following pupariation to downregulate *Ptth* transcription to suppress ecdysone production. Consistent with this, these animals exhibited ecdysone levels similar to (or perhaps lower than) those of controls at pupariation, as reflected by *E75A* transcription, but they displayed increased *E75A* expression 3 h later, potentially because of a failure to downregulate ecdysone production (Fig. 4E; note that in Fig. S2, *E75A* expression was delayed when *EcR* was knocked down constitutively, suggesting that at pupariation in the temperature-shift experiment, *EcR-RNAi* had not had time to affect EcR levels). These data are consistent with a model in which an initial small 20E rise during L3 triggers a positive feedback circuit that generates the metamorphic PTTH/ecdysone surge. Surge-level 20E then suppresses PTTH production following pupariation (Fig. 4F).

## DISCUSSION

### EcR-mediated feedback induces developmental maturation by triggering PTTH neuronal activity

The activation of a neuroendocrine signaling cascade triggers maturation onset in most animals. This activation is associated with body-size gating to ensure the fitness of the reproductive adult. In insects, attainment of ‘critical weight’ during the last larval instar is the main such checkpoint gating the transition to adulthood (Mirth and Riddiford, 2007). After this checkpoint, a larva becomes committed to maturing on a fixed schedule irrespective of further nutrition. Thus, critical weight likely reflects energy stores sufficient to survive the non-feeding maturation process (metamorphosis) and obtain a final adult body size that maximizes fitness (Rewitz et al., 2013). Nutritional status is likewise a main factor permitting the entry into maturation in mammals (Mirth and Riddiford, 2007; Navarro et al., 2007). In humans, body weight correlates with the timing of menarche, which led to the use of the term ‘critical weight’ for the onset of reproductive cycles in humans (Frish and Revelle, 1971). Obese children enter puberty earlier than height-matched non-obese children, and malnutrition and lack of body fat can lead to delayed puberty (Kaplowitz, 2008; Soliman et al., 2014). These observations suggest that the maturation gate reflects not body size per se but rather the amount of body fat, and thus that the neuroendocrine system controlling the timing of this process somehow assesses nutritional and energetic stores. Interestingly, the adipokine leptin regulates pubertal maturation in mammals (Shalitin and Phillip, 2003). Circulating leptin levels correlate with adiposity, and leptin-deficient humans and mice fail to undergo puberty. Leptin may therefore communicate body-fat levels to the neuroendocrine system controlling puberty, which could explain the link between childhood obesity and early onset of puberty. In insect larvae, the fat body is the main nutrient-storage and -sensing organ, releasing numerous nutrient-dependent insulin-regulating hormones (Boulan et al., 2015). Insulin is a stimulator of ecdysone production, thus coupling adipose-tissue nutrient sensing to the neuroendocrine maturation axis in *Drosophila* (Colombani et al., 2005; Mirth et al., 2005). Among the insulinotropic adipokines is Unpaired 2 (Upd2), which is structurally and functionally similar to human leptin. Upd2 acts through the JAK/STAT receptor Domeless (Dome) in GABAergic neurons that regulate insulin secretion from the insulin-producing cells (IPCs) in the brain, which are the primary source of circulating insulin (Rajan and Perrimon, 2012). Thus, related adiposity hormones that signal nutrition and energy storage influence the neuroendocrine events that lead to the onset of maturation in divergent systems.

Mammalian GnRH-producing neurons regulate the timing of puberty onset, and these cells are activated by the neuropeptide KISS1. The PTTHn, activated by the KISS1 ortholog AstA and its receptor AstA-R1 (Deveci et al., 2019), serve this function in *Drosophila*. This suggests conservation of the overall neuroendocrine architecture of the maturation-initiation system. AstA is regulated by nutritional intake, providing another potential link between energy status and maturation onset (Hentze et al., 2015). Furthermore, PTTHn-specific knockdown of *Insulin receptor* (*InR*) or *dome*, encoding the Upd2 receptor, produced size phenotypes in our screen (Table S1), suggesting that the PTTHn integrate systemic nutrition-regulated signals and may also receive input via insulin from the IPCs themselves. Because PTTH controls developmental timing, and insulin is the main growth-regulatory factor, these results suggest that Upd2 may link growth and maturation by coordinating the activity of both the IPCs and the PTTHn. Knockdown of the amino-acid transporters Polyphemus and Minidiscs also induced strong growth effects in our screen, suggesting that the PTTHn may also sense nutrient status autonomously; in the IPCs, Minidiscs is required for inducing insulin secretion after intake of the amino acid leucine (Manière et al., 2016).

This raises the key question of how these nutritional cues lead to the surge mode of GnRH/PTTH release that initiates maturation. Our findings suggest that ecdysone feedback, via EcR in the PTTHn, is the mechanism that induces the PTTH and ecdysone surge towards the end of larval development. This is further reinforced by EcR-mediated positive feedback on ecdysone production in the PG (Moeller et al., 2013). We propose that the triggering event that begins the feedback cycle is a small nutrient-dependent ecdysone peak early in the L3 stage. Nutritional signaling via insulin acts directly on the PG and is required for ecdysone production pre-critical weight but not post-critical weight (Koyama et al., 2014; Shingleton et al., 2005). Furthermore, PTTH secretion is also controlled by nutrition and is required for normal attainment of critical weight (Galagovsky et al., 2018; Shimell et al., 2018), suggesting that PTTH acts together with insulin before attainment of critical weight to generate a small nutrient-dependent rise in ecdysone production at the beginning of L3. This small ecdysone peak upregulates *Ptth* via EcR and, under this scenario, corresponds to critical weight, which occurs ∼10 h after the L2-L3 transition. Thus, when ecdysone reaches the threshold corresponding to critical-weight attainment, it generates an irreversible, self-sustaining feedback activation of the neuroendocrine system by promoting the PTTH surge that triggers the maturation-inducing ecdysone pulse towards the end of L3 (Fig. 4F). This model is supported by findings showing that a small nutrient-sensitive ecdysone peak early in L3 does indeed signal critical weight (Koyama et al., 2014).

The main feature of this model is ecdysone feedback onto the PTTHn via a mechanism requiring EcR in these cells. EcR/Usp may regulate *Ptth* expression by direct binding to the *Ptth* enhancer or through downstream target transcription factors regulated by this complex. Many transcription factors are known to be targets of EcR (King-Jones and Thummel, 2005), and EcR may indirectly regulate *Ptth* expression by altering the expression of one or more of these. Indeed, RNAi against certain known EcR-induced transcription factors, such as *Hr39*, *Hr3* and *ftz-f1*, produced phenotypes in our screen, consistent with a possible role in *Ptth* regulation. Hr3 and Ftz-F1 are also known to participate in ecdysone regulation in the PG (Parvy et al., 2005, 2014), as is another nuclear receptor, Knirps (Danielsen et al., 2014), which was also identified in our screen as a potential regulator of PTTH. Clarifying the precise mechanism by which EcR controls *Ptth* expression will be an interesting topic for future investigation.

### Conserved neuroendocrine circuitry triggers maturation onset

Early maturation is associated with smaller adult size in both flies and humans, as this event limits the juvenile growth period (Carel et al., 2004; Rewitz et al., 2009). The prevalence of precocious puberty has been linked with the increasing rates of childhood obesity; however, the mechanisms that gate GnRH secretion at the time of puberty are poorly understood (Tena-Sempere, 2012). The mammalian HPG axis controlling the onset of puberty is regulated by feedback control in which steroid hormones act to regulate the GnRH-expressing neurons, but whether these neurons themselves are direct steroid targets is still debated (Kaprara and Huhtaniemi, 2018). However, the KISS1-expressing neuronal population has also emerged as a possible link between sex steroids and the GnRH neurons (Dungan et al., 2006).

Many studies in *Drosophila* and other insects have explored the neuroendocrine PTTH-PG-ecdysone axis. The existence of feedback control between ecdysone and PTTH has been hypothesized for decades and is supported by studies of PTTH in other insects, especially in lepidopterans (Hossain et al., 2006; Sakurai, 2005), in which hemolymph titers of PTTH and ecdysone are clearly correlated during the last larval instar (Mizoguchi et al., 2002, 2001). A PTTH surge immediately precedes a rise in ecdysone levels, and gradual increases in ecdysone levels appear to reinforce the peak levels of circulating PTTH, suggesting that ecdysone might positively influence PTTH release. This is supported by findings that injection of ecdysone before an endogenous PTTH peak induces a premature rise in PTTH, whereas injection of ecdysteroid-22-oxidase, a potent enzymatic inactivator of ecdysteroids, inhibits this rise (Mizoguchi et al., 2015). We demonstrate here an EcR-dependent positive-feedback mechanism, operating specifically within the PTTHn, that regulates the transcription of *Ptth*. Our findings show a mechanism by which steroid-mediated feedback signaling triggers the PTTH surge at the onset of metamorphosis, suggesting that feedback control is an evolutionarily conserved regulator of the neuroendocrine signaling that initiates the onset of maturation.

## MATERIALS AND METHODS

### Fly husbandry

All animals were reared on a standard cornmeal diet (Nutri-Fly Bloomington formulation) at 25°C under 12-h light/dark cycle conditions, with 60% relative humidity, unless otherwise stated. Larvae of mixed sex were used in all experiments. The following fly lines were used: *UAS-EcR.gRNA* was generated in this study; *Ptth*-*GAL4, UAS-Dicer-2* and *NP423-GAL4, UAS-Dicer-2* were generous gifts from Pierre Léopold (Institut Curie, Paris, France); *Ptth-GAL4* and *UAS-Ptth::HA* (McBrayer et al., 2007) and *phm-GAL4* (Ono et al., 2006) were kindly provided by Michael O'Connor (University of Minnesota, Minneapolis, MN, USA); *w^1118^* (#60000), *UAS-EcR-RNAi* #1 (#37058), *UAS-EcR-RNAi* #2 (#37059), *UAS-Ptth-RNAi* (#102043) and *UAS-torso-RNAi* (#101154) were obtained from the Vienna *Drosophila* Resource Center (VDRC); *UAS-EcR.B1* (#6469), *UAS-EcR.A* (#6470), *UAS-EcR-RNAi* #3 (#50712) and *UAS-Cas9.P* (#54594) were obtained from the Bloomington *Drosophila* Stock Center (BDSC).

### Fly genetics

To achieve CRISPR/Cas9-mediated disruption of *EcR* under GAL4/UAS control, we generated a UAS construct expressing two *EcR*-targeted gRNAs (below) in the backbone of vector pCFD6 (Port and Bullock, 2016), which was obtained from AddGene (#73915). The gRNA sequences were designed and checked for specificity and efficiency using online tools at http://www.flyrnai.org/crispr/ and http://targetfinder.flycrispr.neuro.brown.edu/. Two sequences with high predicted efficiency and no off-target binding sites were chosen 232 base pairs apart within exon 3 of *EcR*, an exon shared between all *EcR* transcripts that encodes the protein's DNA-binding domain. Efficient induction of double-strand breaks should thus delete the DNA-binding domain and likely introduce frame-shift mutations as well, rendering the locus nonfunctional. Oligonucleotides containing the gRNA sequences were synthesized and inserted into *pCFD6*. The correct *pCFD6-UAS-EcR.gRNA* product was verified by sequencing, and transgenic animals were generated in-house and by Bestgene (Chino Hills, CA, USA). Fly stocks were constructed using standard techniques. gRNA sequence #1: TTCATCGCACATTGGTTCTC; gRNA sequence #2: GCAAGAAGGGACCTGCGCCA.

### Synchronization of development

To synchronize development for timed experiments, parental flies were allowed to lay eggs for 2-4 h on an apple-juice agar plate coated with a thin layer of yeast paste; hours AEL was measured from the midpoint of this time. After 24 h, newly hatched L1 larvae were collected and transferred to vials containing standard food at a density of 30 larvae per vial.

### *Ex vivo* incubation with 20E

Four biological replicates of ten brains with an intact ring gland were dissected from synchronized L3 larvae at 110 h AEL in Schneider's insect medium (Sigma-Aldrich, S0146). The tissue was transferred to Schneider's medium containing 20E (Sigma-Aldrich, H5142) at 0, 5, 50, 500 or 5000 ng/ml and incubated for 6 h at room temperature (roughly 24°C). RNA was then extracted from tissue as described below.

### Real-time quantitative PCR (qPCR) analysis

Four to six biological replicates of five whole larvae or ten dissected brains of each genotype were collected at the indicated times after egg laying (AEL) or puparium formation. Samples were flash-frozen in dry ice and stored at −80°C. The samples were thoroughly homogenized in 350 µl ice-cold lysis buffer containing 1% β-mercaptoethanol, and RNA was extracted using RNeasy mini kit (Qiagen) with DNase treatment according to the manufacturer's instructions. RNA concentrations from whole-larval samples were measured using a NanoDrop spectrophotometer (Thermo Fisher) and adjusted to 300 ng/µl. Synthesis of cDNA from RNA was carried out with a 10 µl RNA sample using a High-Capacity cDNA Transcription Kit (Applied Biosystems). qPCR reactions were performed using the QuantiTect SYBR-Green PCR Kit (Qiagen) on an Mx3005p qPCR system, and transcript levels were normalized against *RpL23*, which is stably expressed across tissues and larval stages (Danielsen et al., 2014). Primers used for this study are listed in Table S2.

### Immunostaining and confocal microscopy

For each genotype, 15 larvae were collected at the indicated times AEL. Each larva was rinsed in water and dissected in cold PBS, and tissues were fixed in 4% formaldehyde in PBS for 30 min, washed in PBS+0.1% Triton X-100 (PBST; one quick rinse followed by three 15-min washes with slow rocking motion), and blocked in PBST+5% normal goat serum (NGS) for at least 1 h. Blocking buffer was exchanged with PBT+5% NGS containing primary antibodies, and tissues were incubated overnight at 4°C. Samples were washed as before and incubated at 4°C overnight with secondary antibodies in PBST. Samples were washed again as above, washed in PBS to remove Triton X-100, and incubated in PBS at 4°C. Brains were mounted on poly-lysine-coated glass slides in ProLong Gold anti-fade reagent (Invitrogen). Fluorescence images were captured using a Zeiss LSM 800 confocal laser scanning microscope coupled with AiryScan technology and were then analyzed using the Fiji software package (https://imagej.net/Fiji). All samples for time-course data were imaged with identical settings. Quantifications of fluorescence intensity were performed by creating summed projections of each individual PTTHn followed by measurements of the anti-PTTH signal in the cell body and the anti-EcR signal in the nuclei using the following formula: integrated density−(area×mean background fluorescence). Mean fluorescence of brain tissue without positive PTTH and EcR signal in each individual projection was subtracted as background for each channel. Guinea pig anti-PTTH was purified using the Melon Gel IgG Spin Purification Kit (Thermo Scientific) from anti-PTTH serum generously provided by Pierre Léopold (Yamanaka et al., 2013); the purified IgG was used at 1:500. Rabbit anti-Phm (1:400) (Ono et al., 2006) was a generous gift of Michael O'Connor. Mouse monoclonal anti-EcR (clone Ag10.2) was obtained from Developmental Studies Hybridoma Bank and was used at 1 µg/ml. Secondary antibodies used were Alexa Fluor 555-conjugated goat anti-mouse, Alexa Fluor 488 goat anti-rabbit and Alexa Fluor 647 goat anti-guinea-pig (Thermo Fisher, A21422, A1108 and A21450), all used at 1:200.

### Ecdysteroid measurements by ELISA

Ecdysteroid levels were measured using a competitive 20-hydroxyecdysone ELISA kit (Bertin Bioreagent Cayman, 501390). Four biological replicates of ten larvae from each genotype were collected at the indicated times AEL. Larvae were washed in water, dried on a Kimwipe, and weighed in groups of ten before they were transferred into empty Eppendorf tubes, flash-frozen on dry ice, and stored at −80°C. Extraction of ecdysone was performed by thoroughly homogenizing the frozen samples in 300 µl ice-cold methanol with a plastic pestle. Samples were centrifuged at 17,000 ***g*** for 10 min, and the supernatant was transferred and split into two Eppendorf tubes, each containing approximately 150 µl supernatant. Methanol from both tubes was evaporated in a vacuum centrifuge for 60 min, and pellets were re-dissolved by adding 200 µl supplied EIA buffer to one of the two Eppendorf tubes. This was vortexed, and the same 200 µl of EIA buffer was transferred to the second tube followed by further vortexing. The ELISA was performed using rabbit anti-20E-coated wells, acetylcholinesterase-conjugated 20E, and serial dilutions of 20E as a standard. In brief, mouse anti-rabbit-coated wells were washed five times with 200 µl EIA buffer. EIA buffer, rabbit anti-20E, acetylcholinesterase-conjugated 20E, and standards/samples were then added to their respective wells, and the plate was covered and incubated at 4°C in darkness overnight. The next day, wells were washed six times with 200 µl EIA buffer, and the activity of the remaining acetylcholinesterase enzyme was quantified by adding Ellman's reagent and reading absorbance at 405 nm every 15 min with an ELx800 plate reader (BioTek).

### Measurement of developmental timing and pupal size

Synchronized larvae were assayed for pupariation timing by noting newly pupated animals at regular time intervals. The time at which 50% of animals had pupariated, P50, was determined by linear extrapolation between scores before and after reaching 50%. To measure pupal size, pupae were mounted on a glass slide, and images were captured with a Chameleon3 camera (FLIR Systems). Images were processed using a custom script (Moeller et al., 2017) in the MATLAB environment (MathWorks). The MATLAB script for quantification of pupal sizes has been published previously (Moeller et al., 2017).

### Statistics

Statistical analysis was performed in Prism software (GraphPad). Statistical differences between a control group and several other groups were analyzed by one-way ANOVA followed by Dunnett's multiple-comparisons tests; the difference between one control group and one other group was analyzed by an unpaired two-tailed Student's *t*-test. Bar graphs show mean±s.e.m. *P*-values are indicated as: *P<0.05, **P<0.01, ***P<0.001.

## Supplementary Material

10.1242/develop.188110_sup1 Supplementary information
