# Supplementary material for: Ecdysone-dependent feedback regulation of prothoracicotropic hormone controls the timing of developmental maturation
Source: Development. 2020 Jul 24;147(14):dev188110. doi: 10.1242/dev.188110 (PMC7390634; doi:10.1242/dev.188110)
Supplement: Supplementary information [file develop-147-188110-s1.pdf]

Supplementary information

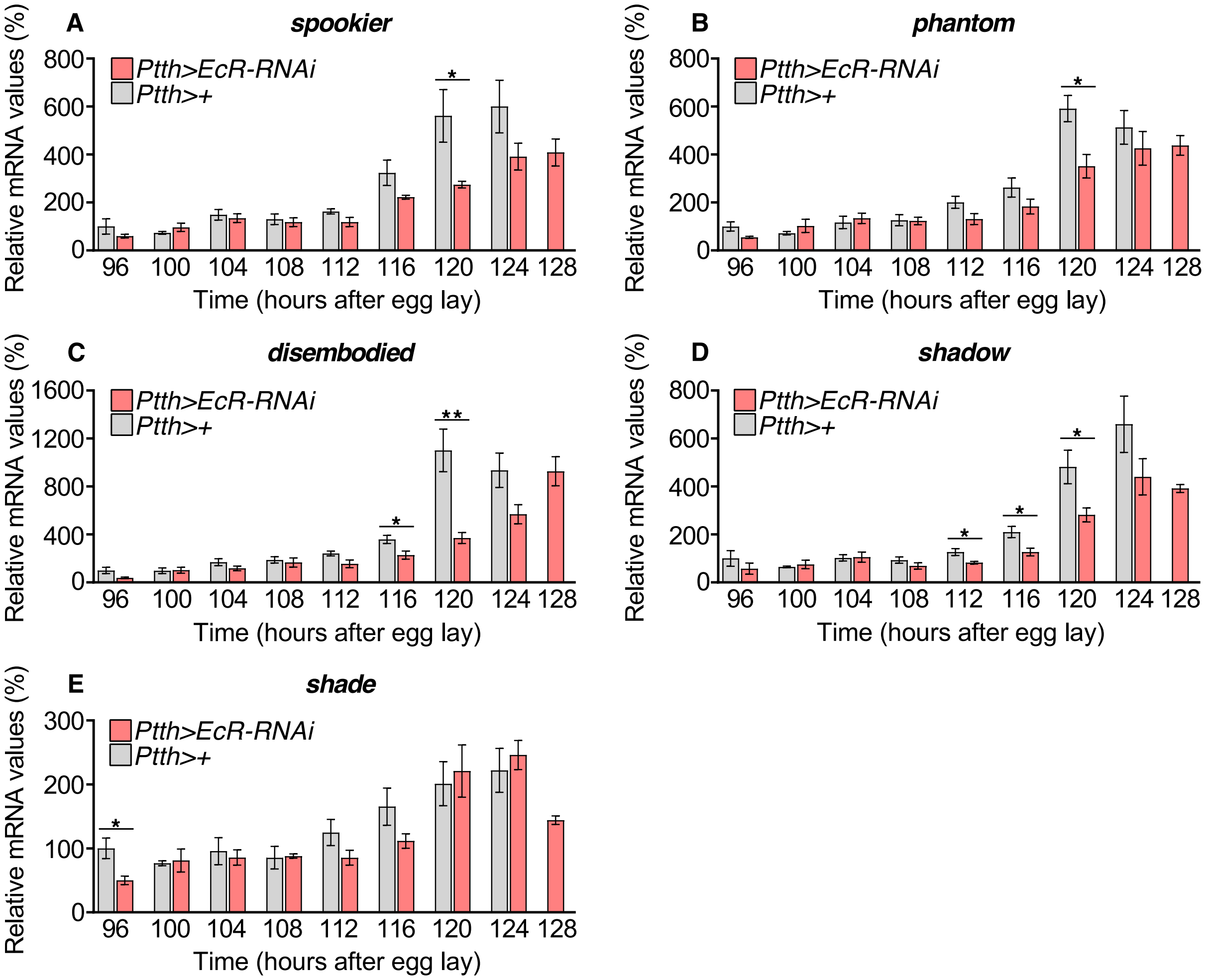

Figure S1. *EcR* knockdown in the PTTHn impairs the expression of ecdysone-biosynthetic genes at the onset of maturation. (A-E) The increase in expression of ecdysone-biosynthetic genes is reduced or delayed when *EcR* is knocked down in the PTTHn. Expression of PG-specific genes *spookier*, *phantom*, *disembodied*, and *shadow* is reduced and delayed in animals with *EcR-RNAi* knockdown the in PTTHn. Expression of *shade* in peripheral tissues is not regulated by PTTH and therefore is unaltered. Statistics: t-test for pairwise comparison; *, $P < 0.05$; **, $P < 0.01$; ***, $P < 0.001$.

Development • Supplementary information

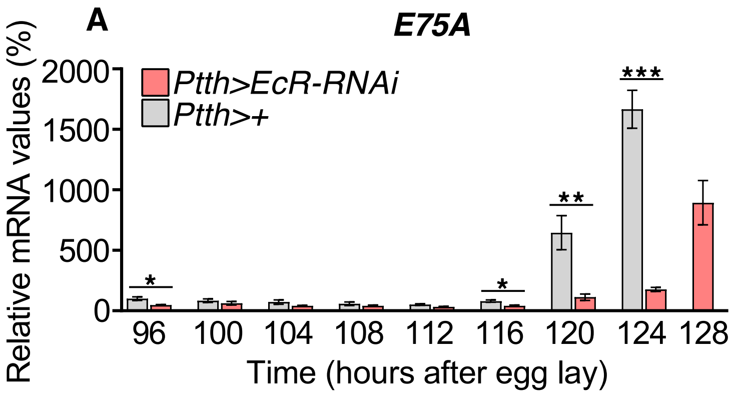

Figure S2. Induction of the ecdysone proxy gene *E75A* is blunted by knockdown of *EcR* in PTTHn. (A) The increase in expression of the ecdysone-inducible gene *E75A* is reduced and delayed in *Ptth>EcR*-knockdown animals compared to controls, indicating impaired ecdysone production. Statistics: Student's t-test for pairwise comparison; *, $P < 0.05$; **, $P < 0.01$; ***, $P < 0.001$.

Development • Supplementary information

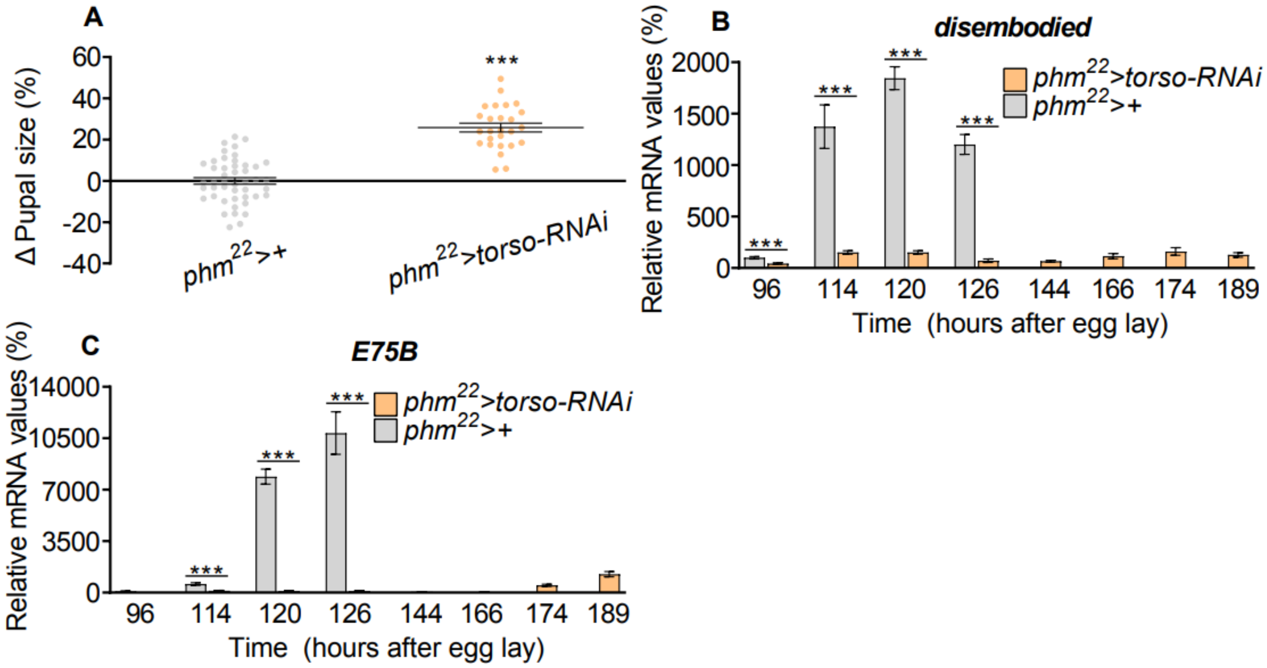

Figure S3. Knockdown of *torso* in the PG has the expected effects on pupal size, *disembodied* expression, and *E75B* ecdysone-proxy transcription (A) RNAi-induced knockdown of *torso*, encoding the PTTH receptor, in the prothoracic gland (PG) using *phm-GAL4* (*phm>*) leads to increased pupal size via prolonged development. (B) The level of the ecdysone-biosynthetic gene *disembodied* does not increase in animals in which *torso* is knocked down in the PG, consistent a lack of ecdysone synthesis (and thus of feedback). (C) Expression of the ecdysone-induced *E75B* proxy gene peaks at 126 hours in control animals, whereas *torso* knockdown greatly reduces and delays this peak, indicating that ecdysone levels are reduced in these animals. Statistics: One-way ANOVA with Dunnett's multiple comparisons or t-test for pairwise comparison; *, P < 0.05; **, P < 0.01; ***, P < 0.001.

Development • Supplementary information

Table S1. Data from the PTTHn RNAi screen

Click here to Download Table S1

Table S2. Primers used for qPCR

**Target gene**	**Forward sequence**	**Reverse sequence**
Ptth	TGAAGGTTTGCACGAGATGG	CTGTGGGATGTGGAGTGCTG
E75A	ACCACAGCACCACCCATTT	TGTTTGGCGGTAGTTTCAGG
E75B	CAACAGCAACAACACCCAGA	CAGATCGGCACATGGCTTT
phm	GGATTTCTTTCGGCGCGATGTG	TGCCTCAGTATCGAAAAGCCGT
spok	TATCTCTTGGGCACACTCGCTG	GCCGAGCTAAATTTCTCCGCTT
dib	TGCCCTCAATCCCTATCTGGTC	ACAGGGTCTTCACACCCATCTC
sad	CCGCATTCAGCAGTCAGTGG	ACCTGCCGTGTACAAGGAGAG
shd	CGGGCTACTCGCTTAATGCAG	AGCAGCACCACCTCCATTTC
ftz-f1	AATCAGCAGCACCACAGCA	TTGGCATTGTTGGCGATATT
RpL23	GACAACACCGGAGCCAAGAACC	GTTTGCGCTGCCGAATAACCAC

Development • Supplementary information
