# Supplementary material for: Ecdysone-dependent feedback regulation of prothoracicotropic hormone controls the timing of developmental maturation
Source: Development. 2020 Jul 24;147(14):dev188110. doi: 10.1242/dev.188110 (PMC7390634; doi:10.1242/dev.188110)
Supplement: Supplementary file 2 [file develop-147-188110_review_history.pdf]

Ecdysone-dependent feedback regulation of prothoracicotropic hormone controls the timing of developmental maturation

Christian F. Christensen, Takashi Koyama, Stanislav Nagy, E. Thomas Danielsen, Michael J. Texada, Kenneth A. Halberg and Kim Rewitz
DOI: 10.1242/dev.188110

Editor: Cassandra Extavour

Review timeline
Original submission: 10 January 2020
Editorial decision: 17 April 2020
First revision received: 20 May 2020
Accepted: 26 June 2020

Original submission

First decision letter

MS ID#: DEVELOP/2020/188110

MS TITLE: Ecdysone-dependent feedback regulation of prothoracicotropic hormone times developmental maturation

AUTHORS: Christian Fokdal F. Christensen, Takashi Koyama, Stanislav Nagy, E. Thomas Danielsen, Michael J. Texada, Kenneth A. Halberg, and Kim Rewitz

I have now received all the referees reports on the above manuscript, and have reached a decision. The referees' comments are appended below, or you can access them online: please go to BenchPress and click on the 'Manuscripts with Decisions' queue in the Author Area.

The overall evaluation is positive and we would like to publish a revised manuscript in Development, provided that the referees' comments can be satisfactorily addressed. Please attend to all of the reviewers' comments in your revised manuscript and detail them in your point-by-point response. If you do not agree with any of their criticisms or suggestions explain clearly why this is so.

We are aware that you may currently be unable to access the lab to undertake experimental revisions. If it would be helpful, we encourage you to contact us to discuss your revision in greater detail. Please send us a point-by-point response indicating where you are able to address concerns raised (either experimentally or by changes to the text) and where you will not be able to do so within the normal timeframe of a revision. We will then provide further guidance. Please also note that we are happy to extend revision timeframes as necessary.

Reviewer 1

Advance summary and potential significance to field

In the manuscript entitled “Ecdysone-dependent feedback regulation of prothoracicotropic hormone times developmental maturation”, Christensen and coworkers showed how ecdysone levels regulate PTTH levels. Although the feedback between PTTH and ecdysone is not an

© 2020. Published by The Company of Biologists under the terms of the Creative Commons Attribution License (https://creativecommons.org/licenses/by/4.0/).

unexpected result, the authors provide solid experiments to demonstrate this control at the level of PTTHn regulating EcR levels.

Comments for the author

Most of the results suggest a delay in reaching the correct levels of PTTH EcR and ecdysone to moult. Is the EcR knockdown completely successful? From figure 2C and D, using CRISPR it seems that yes. However, from figure 3A it seems that the knockdown is not complete.
On the other hand, it is known that if puparation is delayed the animal eats more and becomes bigger. How can be the nutritional signals included in the model proposed by the authors to control PTTH release?
Fig 1D. Is there any difference between the pupal size from PTTH RNAi and EcR RNAi samples? In the manuscript is indicated that the results are similar in both treatments. However, from the figure, it seems that some difference exists comparing pupal size and time of puparation.
All the Halloween genes, in EcR-RNAi individuals, showed a delay in the expression levels. At puparation, are the levels of Halloween genes in treated animals at the same level than in control ones? The expression profile of E75 (E75A and E75B) in these individuals is also delayed, with correspondence with the expression of Halloween genes in treated animals. If there is a delay in the expression, I'm not sure about to write reduction of expression.
Page 5, three lines before the end. Phantom is not exclusive of PG, most of the Halloween genes have been described in insect ovaries. Moreover, the results obtained by immunostaining with an antibody against Phantom are not explained in the text nor in the figure 2C, D.
Page 6. Four lines before the end. Authors wrote, "rapid peptide release presumably underlies the fall in intracellular peptide staining (Fig. 3B)".
It is possible to observe an increase of peptide in the PG to corroborate this suggestion?
Page 6. Last sentence and Fig 3C and 3D. EcR knockdown significantly reduces PTTH levels. However, PTTH levels in treated individuals tend to increase at 120 hours. Comparing the expression levels between 116 hours and 120 hours is this increase statistically significant? How can be explained the increase of ecdysone levels produced in EcR-RNAi individuals at 124h? It is possible to measure ecdysone levels in treated animals beyond 124 AEL perhaps at the moment of puparation?

Particular comments
Fig 1B: revise the use of ",“ to indicate decimals

<u>Reviewer 2</u>

Advance summary and potential significance to field

Summary and Critique
This study investigates the genetic control of metamorphosis in Drosophila; specifically the interplay between prothoracicotropic hormone (PTTH) and Ecdysone Receptor (EcR). Previous studies from this lab and others demonstrated that positive and negative feedback circuits shape the timing and strength of ecdysone biosynthesis at metamorphosis. PTTH up-regulates ecdysone production in prothoracic gland neurons to trigger the larval to pupal transition. While insulin signaling (via DILP8) and circadian rhythms promote PTTH expression, it has been unclear whether other mechanisms promote PTTH expression.

In the current study, Rewitz and colleagues perform an unbiased RNAi screen for regulators of PTTH - specifically, membrane-associated receptors that, upon depletion in prothoracic gland neurons, recapitulate phenotypes associated with loss of PTTH (increased pupal size). The authors find, remarkably, that EcR (and its co-receptor Usp) promotes PTTH transcription. They propose a model in which EcR signaling both promotes PTTH expression and is positively regulated by PTTH via up-regulation of ecdysone biosynthesis.

In general, this is an interesting study that adds a new dimension to our understanding of how steroid hormones regulate developmental transitions. Experiments are well-controlled and use novel reagents for tissue-specific Cas9-mediated EcR knock-out that will be highly beneficial to the field. This short report is appropriate for Development and its readers and is well-written. My

© 2020. Published by The Company of Biologists under the terms of the Creative Commons Attribution License (https://creativecommons.org/licenses/by/4.0/).

specific comments/suggestions for improving the manuscript are detailed below (listed in the order in which they appear in the manuscript).

Comments for the author

Major points
1. Results, pg. 4 (paragraph 1) and Figure 1. The authors use a slightly-biased RNAi screen to identify regulators of PTTH. Interestingly, the two candidates that they chose for further study, EcR and Usp, were not the “top” hits (i.e., the ones that caused the strongest pupal size phenotype, Fig. 1B). This could suggest that EcR/Usp are not direct transcriptional activators of PTTH. It remains formally possible that EcR/Usp indirectly modify PTTH transcription via a downstream effector – potentially another nuclear hormone receptor. The authors should be cautious about over-interpreting their data, as they do not present evidence that EcR/Usp regulation is direct or independent of other potential pathways (such as DILP8 or PDF, mentioned in the Introduction). As such, the model seems a bit over-simplified. A more thorough description of the other hits in the screen could allay these concerns.

2. Results, Figure 2G and Figure 3A-B. The authors compare the levels of PTTH and EcR proteins in different genetic backgrounds and depict the results as a “normalized fluorescence”. However, it some images, it looks as though there is variability between samples (see Figure 3A timepoints 116, 124, and 128). The authors should more clearly explain in the results and methods how immunofluorescence images were quantified, normalized against expression in other parts of the tissue, or somehow standardized between individuals and/or individual cells (and how many individuals were scored).

Reviewer 3

Advance summary and potential significance to field

The juvenile/growth phase of animal development ends with the onset of maturation/puberty and this developmental transition is in turn regulated by a neuroendocrine axis that appears to be conserved between humans and flies. In flies, the core of this neuroendocrine axis involves neurons that secrete PTTH and the prothoracic glands that secrete the steroid hormone ecdysone. It is well known that a surge of ecdysone triggers the onset of maturation; however, the mechanisms that regulate this ecdysone surge are less clear. In this manuscript, the authors provide new insights into how positive and negative feedback "loops" within the neuroendocrine axis generate the timing and profile of the ecdysone surge. As the authors demonstrate, low levels of ecdysone stimulate production of ecdysone through the axis, whereas high levels of ecdysone suppress the production of ecdysone through the axis. Thus, in this model, an initial small pulse of ecdysone can be amplified to generate a massive rise of ecdysone levels, with the resulting high levels triggering the end of the surge. As the authors point out, these steroid-dependent feedbacks are also known to regulate the neuroendocrine axis in mammalian maturation.

Comments for the author

This manuscript provides a nice genetic dissection of a critical neuroendocrine axis that triggers the onset of maturation in flies. The authors' conclusions are based on meticulously executed and controlled experiments. The experiments take full advantage of the genetic toolkit in Drosophila, with tissue-specific, temporal-specific, and even ex vivo approaches to test the proposed model. The manuscript is also well-written and well-organized making it a pleasure to read.

This reviewer does not have any experimental concerns with the manuscript. The only issue, albeit relatively minor relates to the stated novelty of the work. Some of the generalities of this endocrine axis were already known; however the authors provide new mechanistic insights of its "inner" workings, adding potentially significant new details. Toning down the rhetoric on the conservation of the axis, which somewhat implies that it is being discovered with this work would go a long way to placing the contribution of this work into a proper context.

© 2020. Published by The Company of Biologists under the terms of the Creative Commons Attribution License (https://creativecommons.org/licenses/by/4.0/).

First revision

Author response to reviewers' comments

Reviewer #1
The reviewer finds that our work provides solid evidence for feedback regulation of PTTH and we are grateful for this positive appraisal.

1) The reviewer raises the question of whether EcR knockdown is as completely successful as the CRISPR shown in Figure 2C and 2D, since weak EcR stain is still detected in Figure 3A. It is true that RNAi usually reduces expression without eliminating it as would be expected from CRISPR-induced deletion of the locus. Gene knockdown in the PTTH-producing neurons (PTTHn) in particular has been notoriously difficult, due in part to a lack of strong, specific drivers (the Ptth-GAL4 construct is somewhat weak). To overcome these problems, we and others have used two copies of Ptth-GAL4 combined with two copies of UAS-Dicer-2 to enhance the RNAi effect (Deveci et al., 2019). We show in Figure 2A that RNAi-mediated knockdown of EcR delays pupariation similarly to knockdown of Ptth. Neither case can be expected to be a complete loss of function. We therefore developed reagents for tissue-specific CRISPR, which (as also pointed out by Reviewer #2) will be highly beneficial to the field. In any case, we have shown with several RNAi lines that we can induce sufficient knockdown to produce developmental-delay and larval-overgrowth phenotypes, as well as reduced expression of EcR. However, to address the point raised by the reviewer, we have now mentioned the RNAi effect versus the complete elimination (CRISPR knockout) in the Results section of the revised manuscript.

2) The reviewer asks how nutritional signals might be included in the proposed model. We thank the reviewer for pointing out that more focus on how nutritional signals fit in would improve the manuscript. We have now added a paragraph discussing this: Nutritional signaling via insulin acts directly on the PG and is required for ecdysone production before critical weight but not afterwards (Koyama et al., 2014; Shingleton et al., 2005). Furthermore, PTTH secretion is also controlled by nutrition and is required for normal attainment of critical weight (Galagovsky et al., 2018; Shimell et al., 2018), suggesting that PTTH acts together with insulin before critical weight to generate a small nutrient-dependent rise in ecdysone production in the beginning of L3. This small ecdysone peak would upregulate Ptth via EcR and, under this scenario, correspond to critical weight, which occurs ~10 hours after the L2-L3 transition. Thus, when ecdysone reaches the threshold corresponding to critical-weight attainment, it generates an irreversible, self-sustaining feedback activation of the neuroendocrine system by promoting the PTTH surge that in turn triggers the maturation-inducing pulse of ecdysone towards the end of L3. To address the point, we have now included the above discussion in the revised manuscript, including references to articles showing that insulin and PTTH mediate nutrient-dependent ecdysone production, and we have alluded to this nutritional signaling in the model shown in Figure 4F.

3) The reviewer asks whether Ptth-RNAi and EcR-RNAi cause similar effects on pupal size and timing (Figure 1D). The knockdown of Ptth with the NP423-GAL4 driver seems to produce a slightly stronger effect than knockdown of EcR, which could potentially be explained by their different temporal requirements. Ptth mutants exhibit delayed development and increased critical weight, suggesting that PTTH is required (as discussed above in response to point #2) before critical weight to generate the small early-L3 ecdysone peak. Our work suggests that EcR is required in the PTTHn to respond to this increase in ecdysone by activating the PTTH surge after critical weight, which leads to a self-sustaining feedback activation of PTTH and thus of the maturation-inducing neuroendocrine system. Since PTTH is required both before and after critical weight, it is likely that the effect of loss of PTTH on size is greater than that of EcR loss in the PTTHn. To address the point raised by the reviewer, we have changed our wording, so that it does not indicate that they are similar in both treatments.

4) The reviewer raises the question of whether expression of the Halloween genes and E75A/B is only delayed or also reduced. In the manuscript, we write that the Halloween genes are not properly upregulated, which is correct in any case. For E75, we say that its expression is reduced and delayed. To support this claim, which is correctly queried by the reviewer, we have now compared the highest observed E75 levels in controls around the time of pupariation versus the

© 2020. Published by The Company of Biologists under the terms of the Creative Commons Attribution License (https://creativecommons.org/licenses/by/4.0/).

highest levels in knockdown animals around the timing of pupariation. This analysis is now included in the revised Figure 3E, supporting that in fact expression of E75 is both reduced as well as delayed. Other reports have indicated that longer-term exposure to lower levels of ecdysone can trigger responses similar to those seen with higher levels and can even induce pupariation (Moeller et al., 2013). Thus, animals with EcR knockdown in the PTTHn presumably never reach the same ecdysone levels as in controls, but this level is still sufficient if integrated over time to trigger pupariation.

5) We have now omitted the sentence about Phantom's being PG-specific and described in the text that the Phantom stain was used to label the PG. We believe this addresses the point.

6) The reviewer asks whether it is possible to observed an increase of PTTH peptide in the PG to corroborate the suggestion that rapid peptide release presumably underlies the fall in intracellular peptide staining. To our knowledge it is not possible to detect PTTH bound to Torso on the PG surface, so we have omitted this suggestion from the text in the revised version to address this point.

7) The reviewer asks if comparing PTTH levels in control to RNAi knockdown between 116 hours and 120 hours is significant. It is significant between the 120-hour samples, but not when comparing 120 hours to 116 hours. However, even at 128 hours, PTTH expression in the EcR-RNAi has not reached the levels seen earlier in the control, clearly suggesting that PTTH is reduced. The next question is how ecdysone levels eventually can increase in the EcR-RNAi animals. There are several things that can explain this. First of all, there is positive feedback in the PG itself, which may eventually drive ecdysone production up even in the absence of PTTH signaling. In fact, this feedback is mediated by EcR (Moeller et al., 2013) and has recently been shown to involve EGF signaling in the PG through the MAPK pathway (Cruz et al., 2020), the same pathway through which PTTH acts to stimulate ecdysone production. Thus, the PG EGFR feedback circuit would lead to increased MAPK signaling even in the absence of PTTH. Furthermore, during the prolonged L3 stage exhibited by EcR-RNAi animals, increased nutrient signaling in the PG mediated by insulin and TOR may eventually help drive ecdysone biosynthesis up. Regarding the possibility of measuring ecdysone in RNAi animals beyond 124 hours, while we thank the reviewer for bringing this up, we believe that the E75 expression in Figure 3E provides sufficient support for reduced ecdysone levels beyond the 124-hour time point. Moreover, the important point is not so much that ecdysone does not go up in the RNAi animals, but more that the increase is delayed. Even Ptth null mutants do eventually pupariate, indicating that ecdysone signaling does rise without PTTH activity (Shimell et al., 2018). We believe that many lines of evidence support the conclusion that ecdysone signaling is reduced and delayed in the EcR-RNAi animals, including E75 expression, Halloween-gene expression, Ptth expression and peptide levels, and even the fact that Ptth expression provides rescue of EcR loss-of-function.

8) We thank the reviewer for bringing this to our attention. We have now used "." to indicate decimals.

Reviewer #2
The reviewer has offered kind and constructive comments for improving our manuscript, for which we are grateful. We address them below.

1) The reviewer is of course correct to be interested in other hits identified in the screen. We have added a brief discussion of notable nutrition-related hits such as the insulin receptor (InR) and the Unpaired-2 (Upd2) JAK/STAT receptor Domeless that represent potentially interesting avenues for investigation. The adipokine Upd2 regulates insulin secretion from the insulin-producing cells (IPCs) in the brain, which is the primary source of circulating insulin in Drosophila (Rajan and Perrimon, 2012). While PTTH controls developmental timing, insulin is the main growth-regulatory factor, suggesting that perhaps Upd2 coordinates growth and maturation by effects on both insulin and PTTH. Furthermore, the effect of InR knockdown in the PTTHn suggest that they potentially receive direct input from the IPCs via insulin to further coordinate the insulin and PTTH activity during development. Furthermore, knockdown of the amino-acid transporter Minidiscs, which allows direct sensing of leucine in the IPCs (Maniere et al., 2016), produced phenotypes in our screen, suggesting potential cell-autonomous nutritional sensing in the PTTHn as well. We have also added a discussion of the potential for indirect effects downstream of EcR:Usp signaling, including the cascade of

© 2020. Published by The Company of Biologists under the terms of the Creative Commons Attribution License (https://creativecommons.org/licenses/by/4.0/).

ecdysone-induced transcription factors such as Hr3, Hr39, and FTZ-F1; these were identified in our screen, and some of them also regulate ecdysone production in the prothoracic gland (Parvy et al., 2005; Parvy et al., 2014). We also identified another nuclear receptor, Knirps, which regulates ecdysone production in the prothoracic gland (Danielsen et al., 2014), as a potential regulator of PTTH. These hits open interesting avenues for future investigatuions into the precise mechanisms by which EcR controls Ptth expression.

2) The reviewer requests more information about the image-analysis methods used in Figures 2G, 3A, and 3B. To provide more methodological and statistical detail to the reader, we have added more explanatory text in the Methods section and the figure legends, and for each data point in the three figure panels we have indicated the number of neurons measured.

Reviewer #3
We thank the reviewer for making generous comments about the importance and execution of our work. Below we explain how we have addressed the minor issue raised by the reviewer.

1) We have now toned down in both the abstract and throughout the text the rhetoric implying the conservation of the axis being discovered with our work. In the abstract, we now say that our work suggests an overall conservation at the level of the feedback circuitry, rather than of the axis itself. Furthermore, at the end of the introduction and at end of the discussion, we have revised the manuscript to say that our data suggest that “neuroendocrine feedback control” rather than the “neurocircuitry” of developmental maturation is evolutionarily ancient. These changes have improved the manuscript and clarified that our study shows the existence of feedback regulation of the neuroendocrine axis and not the axis itself, which we believe addresses the point raised by the reviewer.

We thank the three reviewers for their helpful comments, which have helped us to improve our manuscript. We hope that our paper, with these revisions, will now meet with your full approval.

If you have any questions, please do not hesitate to contact me.

References

Cruz, J., Martin, D. and Franch-Marro, X. (2020). Egfr Signaling Is a Major Regulator of Ecdysone Biosynthesis in the Drosophila Prothoracic Gland. Curr Biol.
Danielsen, E. T., Moeller, M. E., Dorry, E., Komura-Kawa, T., Fujimoto, Y., Troelsen, J. T., Herder, R., O'Connor, M. B., Niwa, R. and Rewitz, K. F. (2014). Transcriptional Control of Steroid Biosynthesis Genes in the Drosophila Prothoracic Gland by Ventral Veins Lacking and Knirps. PLoS Genet 10, e1004343.
Deveci, D., Martin, F. A., Leopold, P. and Romero, N. M. (2019). AstA Signaling Functions as an Evolutionary Conserved Mechanism Timing Juvenile to Adult Transition. Curr Biol 29, 813-822 e814.
Galagovsky, D., Depetris-Chauvin, A., Maniere, G., Geillon, F., Berthelot-Grosjean, M., Noirot, E., Alves, G. and Grosjean, Y. (2018). Sobremesa L-type Amino Acid Transporter Expressed in Glia Is Essential for Proper Timing of Development and Brain Growth. Cell reports 24, 3156-3166 e3154.
Koyama, T., Rodrigues, M. A., Athanasiadis, A., Shingleton, A. W. and Mirth, C. K. (2014). Nutritional control of body size through FoxO-Ultraspiracle mediated ecdysone biosynthesis. Elife 3.
Maniere, G., Ziegler, A. B., Geillon, F., Featherstone, D. E. and Grosjean, Y. (2016). Direct Sensing of Nutrients via a LAT1-like Transporter in Drosophila Insulin-Producing Cells. Cell reports 17, 137-148.
Moeller, M. E., Danielsen, E. T., Herder, R., O'Connor, M. B. and Rewitz, K. F. (2013). Dynamic feedback circuits function as a switch for shaping a maturation-inducing steroid pulse in Drosophila. Development 140, 4730-4739.
Parvy, J. P., Blais, C., Bernard, F., Warren, J. T., Petryk, A., Gilbert, L. I., O'Connor, M. B. and Dauphin-Villemant, C. (2005). A role for betaFTZ-F1 in regulating ecdysteroid titers during post-embryonic development in Drosophila melanogaster. Dev Biol 282, 84-94.
Parvy, J. P., Wang, P., Garrido, D., Maria, A., Blais, C., Poidevin, M. and Montagne, J. (2014). Forward and feedback regulation of cyclic steroid production in Drosophila melanogaster. Development 141, 3955-3965.
Rajan, A. and Perrimon, N. (2012). Drosophila cytokine unpaired 2 regulates physiological homeostasis by remotely controlling insulin secretion. Cell 151, 123-137.

© 2020. Published by The Company of Biologists under the terms of the Creative Commons Attribution License (https://creativecommons.org/licenses/by/4.0/).

Shimell, M., Pan, X., Martin, F. A., Ghosh, A. C., Leopold, P., O'Connor, M. B. and Romero, N. M. (2018). Prothoracicotropic hormone modulates environmental adaptive plasticity through the control of developmental timing. Development 145.
Shingleton, A. W., Das, J., Vinicius, L. and Stern, D. L. (2005). The temporal requirements for insulin signaling during development in Drosophila. PLoS Biol 3, e289.

Second decision letter

MS ID#: DEVELOP/2020/188110

MS TITLE: Ecdysone-dependent feedback regulation of prothoracicotropic hormone times developmental maturation

AUTHORS: Christian Fokdal F. Christensen, Takashi Koyama, Stanislav Nagy, E. Thomas Danielsen, Michael J. Texada, Kenneth A. Halberg, and Kim Rewitz
ARTICLE TYPE: Research Article

With many apologies for the delay, I am happy to tell you that your manuscript has been accepted for publication in Development, pending our standard ethics checks.

Reviewer 2

Advance summary and potential significance to field

This study that adds mechanistic data supporting the feedback between PTTH and EcR, adding a new dimension to our understanding of how steroid hormones regulate developmental transitions. Development of novel reagents for tissue-specific Cas9-mediated EcR knock-out will also be highly beneficial to the field.

Comments for the author

The authors have addressed all three reviewers' concerns, including my own. I recommend for publication.

Reviewer 3

Advance summary and potential significance to field

In this manuscript, the authors provide new insights into how positive and negative feedback "loops" within the neuroendocrine axis generate the timing and profile of the ecdysone surge at the onset of Drosophila metamorphosis.

Comments for the author

The authors have satisfactorily addressed this reviewer's comments.

© 2020. Published by The Company of Biologists under the terms of the Creative Commons Attribution License (https://creativecommons.org/licenses/by/4.0/).
